# Supplementary material for: Transcriptome analysis reveals temporally regulated genetic networks during Drosophila border cell collective migration
Source: BMC Genomics. 2023 Dec 1;24:728. doi: 10.1186/s12864-023-09839-8 (PMC10693066; doi:10.1186/s12864-023-09839-8)
Supplement: Supplementary file 1 — Supplementary Material 1 [file 12864_2023_9839_MOESM1_ESM.pdf]

Supplementary Materials: Burghardt et al.

Supplemental Figures, Supplemental Figure Legends, Supplemental Table, and Supplementary Data Information

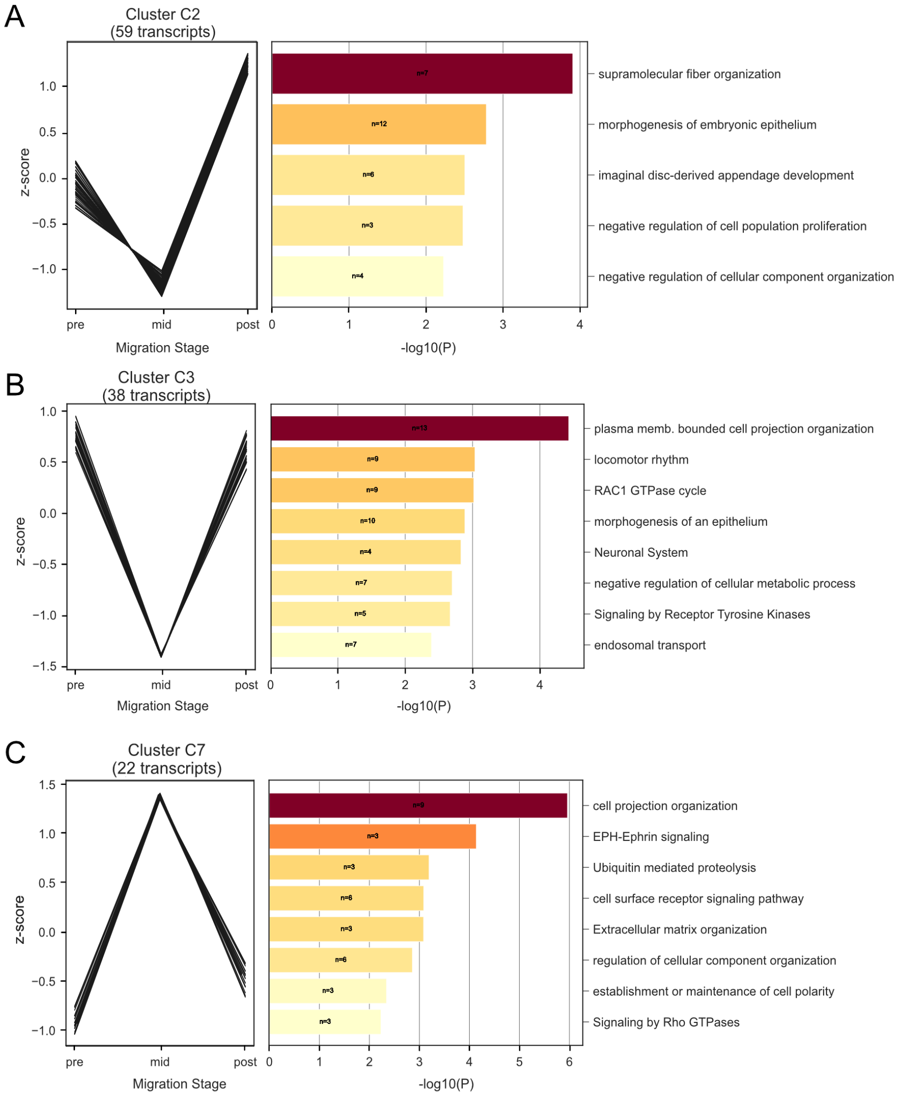

Supplemental Figure 1. Differentially co-expressed transcripts are up- and down-regulated in border cells at mid-migration and enriched for shared biological function.
(A-C, left graphs) Significantly differentially expressed transcripts sorted by clust into shared co-expression patterns in pre-, mid-, and post-migration stages. (A-C, right graphs) Metascape pathway and process enrichment analysis results for each co-expression cluster, showing the mostly significantly enriched terms. N, number of genes enriched for a given annotation term; bars show significance of annotation terms, sorted by p values (-log10P; darker color, more significant values). Note that genes can be found in multiple Metascape annotation categories. Cluster C2 (A), C3 (B), and C7 (C) share patterns of stage-specific increased or decreased expression at mid-migration stages. All data are shown in **Supplemental Data 4 and Supplemental Data 5**.

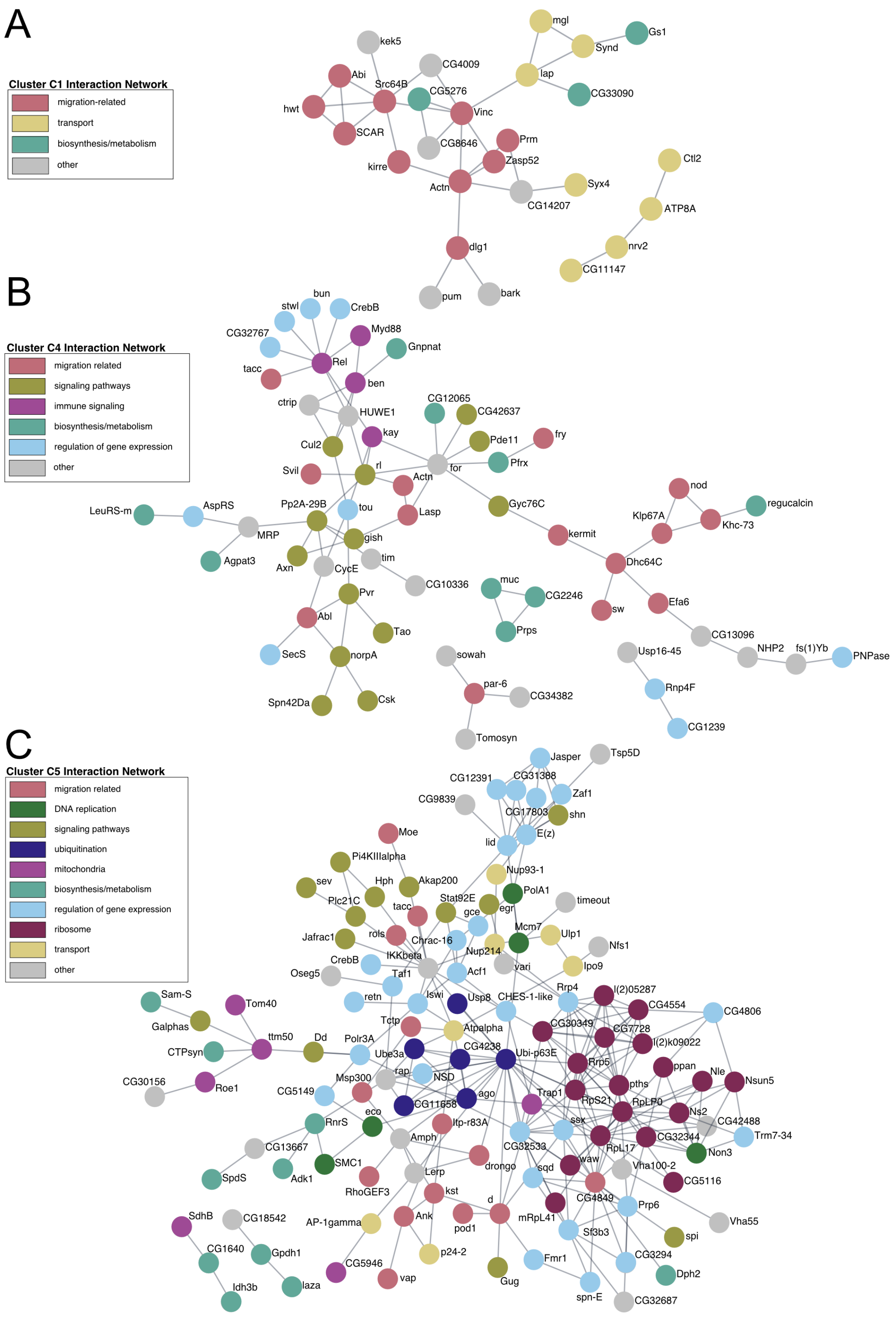

Supplemental Figure 2. Protein-protein interaction networks for co-expression clusters C1, C4 and C5.

(A-C) Physical PPI network analysis of genes in selected co-expression clusters C1 (A), C4 (B) and C5 (C). Individual small networks are found in each cluster in addition to the larger networks. (A) Network of C1 co-expressed genes with expression that increased during border cell migration. (B, C) Networks of C4 (B) and C5 (C) co-expressed genes with expression patterns that decrease during border cell migration. Functional annotation keywords (**Supplemental Data 6**) were used to assign color to proteins in the networks. (A) Co-expression cluster C1 forms a network with migration-related functions (pink). An individual node (top right) and network (lower right) have transport-related functions (mustard). (B) The co-expression cluster C4 network forms nodes with immune signaling (purple) and regulation of gene expression (light blue, upper left), biosynthesis/metabolism (cyan, center), and signaling pathways (green, center). (C) Co-expression cluster C5 forms a large node for ribosome function (magenta, right), nodes for regulation of gene expression (light blue, top, center, and lower right), and a node for signaling pathways (green, upper left).

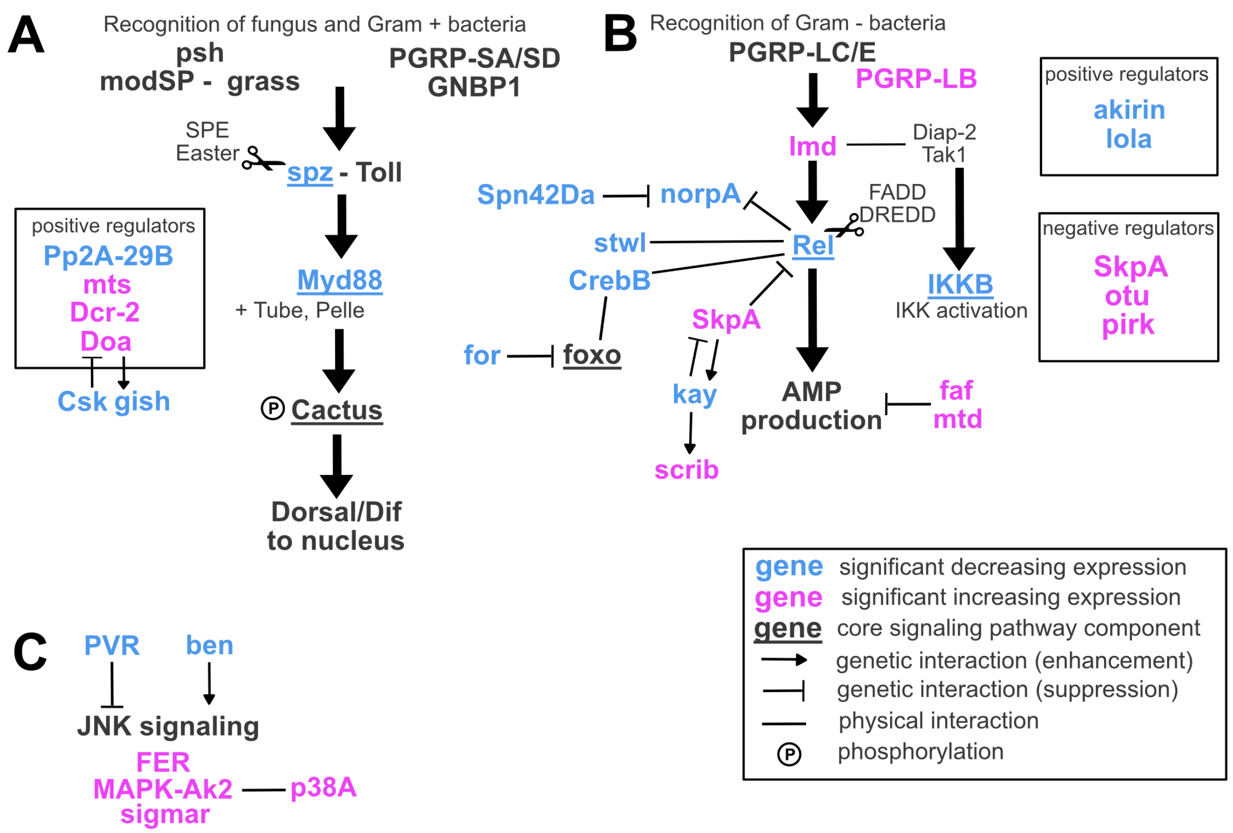

Supplemental Figure 3. Toll, Imd, and JNK signaling pathway genes differentially expressed during border cell migration.
Diagrams of key components and regulators of the Toll (A), Imd (B), and JNK (C) signaling pathways, some of which are differentially expressed during border cell migration. Genes that are differentially expressed in border cells are in bold text, while those that increase in expression are shown in magenta and those that decrease in expression are shown in blue. Genes denoted as core signaling pathway components on FlyBase are underlined. Annotation data is available in **Supplemental Data 7** file.

Supplemental Table 1. Alignment of sequencing reads to the *Drosophila melanogaster* reference genome.

Sample	Total reads available	Number of unalignable reads	Percent unalignable reads	Number of alignable reads	Percent alignable reads	Number of filtered reads due to too many alignments	Percent filtered
Post_1	11541409	1030306	8.93	10502207	91.00	8896	0.08
Post_2	14202132	1156696	8.14	13039017	91.81	6419	0.05
Post_3	14129476	1364205	9.66	12757191	90.29	8080	0.06
Mid_1	12038162	1358990	11.29	10670239	88.64	8933	0.07
Mid_2	14770875	1264871	8.56	13499186	91.39	6818	0.05
Mid_3	13548544	1218221	8.99	12322675	90.95	7648	0.06
Pre_2	13899400	1221410	8.79	12672490	91.17	5500	0.04
Pre_3	15271968	1109436	7.26	14151329	92.66	11203	0.07

Alignment of the sequencing reads to the *Drosophila melanogaster* reference genome for each sample (pre-migration: “Pre_#”; mid-migration: “Mid_#”; post-migration: “Post_#”). All samples were acquired as three biological replicates except pre-migration, which had one replicate fail due to low levels of RNA in the sample.

Supplementary Data Information

Supplemental Data 1. Excel file with raw transcript quantification data in border cells during their migration.

Supplemental Data 2. Excel file showing significantly differentially expressed genes during border cell migration based on quantile normalized genome-wide gene expression with hierarchical clustering.

Supplemental Data 3. Excel file that contains 8 tabs, each detailing genes identified in individual gene classes of interest that were differentially expressed during border cell migration, based on quantile normalized genome-wide expression with hierarchical clustering. Classes of interest include border cell migration genes (tab 1), genes identified in Borghese et al. or Wang et al. microarrays (tabs 2-3), genes enriched for adhesion, epithelial-to-mesenchymal transition, or oogenesis functions (tabs 4-7, respectively), and transcription factors (tab 8).

Supplemental Data 4: Excel file showing all significantly differentially expressed genes during border cell migration based on *clust* expression patterns. Data for co-expression clusters C0-C8 are displayed in tab 1.

Supplemental Data 5. Excel file, with 9 tabs, showing Metascape annotation for genes significantly differentially expressed during border cell migration in an individual co-expression cluster. Data for co-expression clusters C0-C8 are displayed in tabs 1-9, respectively.

Supplemental Data 6. Excel file, with 6 tabs, showing STRING and functional annotation for Cytoscape protein interaction networks for analyzed co-expression clusters. Data for clusters C0, C1, C4, C5, C6, and C8 can be found in tabs 1-6, respectively.

Supplemental Data 7. Excel file showing significantly differentially expressed genes enriched for immune function, with annotations.

Supplemental Data 8. Excel file showing significantly differentially expressed genes enriched for ribosomal function, with annotations, including GO terms.

Supplemental Data 9. Excel file, with 10 tabs, detailing the significantly differentially expressed genes identified in the Dresden Ovary Table (DOT). A summary of the available DOT data, including annotations for stage and cell type-specific expression, are provided for co-expression clusters C0-C8 (tabs 1-9, respectively). Tab 10 contains a summary of the available DOT data for genes with identified border cell-specific expression.

Supplemental Data 10. Excel file, with 4 tabs, detailing the genes that were chosen for the RNAi screen testing function of selected differentially expressed genes in border cells (tab 1). Tab 2 details the RNAi reagents used. The raw migration defect data are shown in tab 3, and the summary of significant migration defect data is in tab 4.
